# Supercritical Carbon Dioxide-Assisted Process for Well-Dispersed Silicon/Graphene Composite as a Li ion Battery Anode

**DOI:** 10.1038/srep32011

**Published:** 2016-08-18

**Authors:** Sang Ha Lee, Sengyoen Park, Min Kim, Dohyeon Yoon, Chalathorn Chanthad, Misuk Cho, Jaehoon Kim, Jong Hyeok Park, Youngkwan Lee

**Affiliations:** 1 School of Chemical Engineering, Sungkyunkwan University, 440-746 Suwon, Korea; 2 School of Mechanical Engineering, Sungkyunkwan University, 440-746 Suwon, Korea; 3 Department of Chemical and Biomolecular Engineering, Yonsei, 120-749 Seoul, Korea

## Abstract

The silicon (Si)/graphene composite has been touted as one of the most promising anode materials for lithium ion batteries. However, the optimal fabrication method for this composite remains a challenge. Here, we developed a novel method using supercritical carbon dioxide (scCO_2_) to intercalate Si nanoparticles into graphene nanosheets. Silicon was modified with a thin layer of polyaniline, which assisted the dispersion of graphene sheets by introducing π-π interaction. Using scCO_2_, well-dispersed Si/graphene composite was successfully obtained in a short time under mild temperature. The composite showed high cycle performance (1,789 mAh/g after 250 cycles) and rate capability (1,690 mAh/g at a current density of 4,000 mA/g). This study provides a new approach for cost-effective and scalable preparation of a Si/graphene composite using scCO_2_ for a highly stable lithium battery anode material.

Silicon (Si) is considered one of the most promising candidates for anode material for lithium ion batteries because of its high theoretical capacity (4,200 mAh/g); however, the material undergoes large volume changes (>300%) upon charge-discharge cycling, resulting in structural collapse and poor electrical contacts within the anode structure, leading to drastic capacity fading^1^,^2^,^3^,^4^,^5^,^6^,^7^. To address this issue, Si anodes have been modified with graphene, the excellent physical properties of which enables this material to assume the role of a buffering matrix to accommodate the volume change of the Si anode and accordingly suppress structural changes while maintaining good electrical conductivity^8^,^9^,^10^,^11^,^12^,^13^,^14^.

However, there are problems associated with graphene composite systems. Mechanical mixing is generally applied for composite preparation; however, it is difficult to ensure a uniform distribution of the Si-based particles over the surface of the graphene due to the insufficient interpenetration capability within the stacked graphene structure^15^,^16^,^17^,^18^. Therefore, to form homogeneous composite, high-energy ball milling for an extended period of time is required^15^. Solution-based complexation approaches using graphene oxide (GO) have also been employed. GO has a negative charge resulting from ionization of its carboxylic acid and phenolic hydroxyl groups, which could exert electrostatic attraction with positively charged materials. To achieve such attraction, some researchers have tried to modify the surfaces of Si particles with surfactant, polymer, or silane^15^,^19^,^20^,^21^. Zhou *et al*. prepared Si/reduced graphene oxide composite using PDDA-modified Si and obtained a specific capacity of 1205 mAh/g after 150 cycles^19^. Miroshnikov *et al*. used 3-aminopropyltriethoxysilane to modify the Si surface for further reaction with epoxy and carboxyl groups present in GO. The prepared Si/reduced graphene oxide composite showed a specific capacity of 1,600 mAh/g after 50 cycles^21^. In these studies, Si/graphene composite was obtained using electrostatic interactions; however, complex pretreatment and post-thermal treatment would be required to modify the Si and to reduce the graphene oxide and/or remove the residual moieties, respectively. Moreover, the precursor of the composite is limited to GO, because a negative charge is necessary for electrostatic interaction. GO is generally prepared through a laborious oxidation process using strong oxidant over a long period of time, which restricts the application due to expense and limited scale. Thus, the development of an inexpensive, scalable, and convenient process to prepare Si/graphene composite is required.

Recently, supercritical fluid has been used in many studies to prepare graphene or graphene composite because of its gas-like diffusivity, liquid-like density, low viscosity, and zero surface tension^22^. In particular, supercritical carbon dioxide (scCO_2_) has attracted sustained interest in both the scientific and industrial fields because of its low critical temperature and pressure, as well as it being nonflammable, nontoxic, inexpensive, and environmentally benign^23^. Researchers have prepared few-layer graphene using scCO_2_, because it can easily penetrate graphite, similar to gas, and stabilized the graphene by preventing aggregation. However, the graphene is easily aggregated through van der Waals interactions after depressurization, and agents such as polymers or surfactants should be used to prevent the formation of covalent bonds between the graphene^24^. Researchers have also tried to prepare graphene/metal, graphene/metal oxide, and graphene/polymer composite using scCO_2_ fluid^25^,^26^ and obtained a graphene composite decorated with small, homogenous, and well-distributed particles. Akbarinezhad *et al*. reported a graphite and PANi composite prepared using scCO_2_, in which PANi particles were homogenously deposited between graphite layers with aid of the π-π interactions between PANi chains and graphite layers^27^. In these studies, various metal precursors or monomer were used for the reaction under scCO_2_ fluid; however, to the best of our knowledge, few studies have tried to prepare Si/graphene composite, which might be due to highly reactive nature of Si precursors.

Here, we demonstrate a strategy for fabricating Si/graphene composite using Si nanoparticles. With the aid of scCO_2_, we obtained a homogenous composite under a mild condition in a short time. We used graphene nanoplatelets instead of GO, which were prepared without an oxidation process, providing the advantages of lower cost and higher stability due to the absence of defects^28^,^29^. Si nanoparticles were modified with a thin layer of polyaniline (PANi) by simple chemical polymerization before using scCO_2_ in order to deposit the nanoparticles on the graphene sheets through the π-π interaction between the graphene and PANi shell. The composite structure was fully characterized, and its electrochemical performance as a lithium battery anode material was carefully analyzed.

## Results

**Figure 1:**
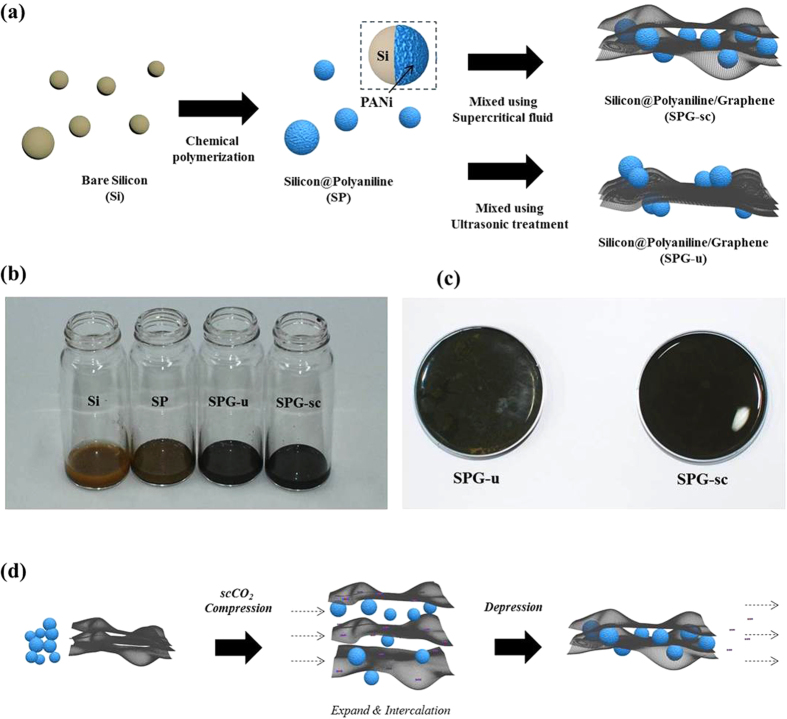
(**a**) Scheme for preparation of the composite, (**b,c**) photographs of the prepared solution. (**d**) Scheme for the mechanism of SPG-sc fabrication.

Figure 1a illustrates the strategy for the fabrication of the Si/graphene composite. Si nanoparticle was first covered by PANi via a simple chemical polymerization. To prepare the composite, the PANi-coated Si nanoparticles (SP) and graphene were dispersed in an ethanol solvent. The solution was mixed with scCO_2_ with stirring and then dried to prepare the composite powder (SPG-sc). For comparison, the solution was mechanically mixed using an ultrasonication instrument (SPG-u) instead of scCO_2_. Graphene contents were varied for the initial optimization process; based on these results, graphene content was set at 30 wt% for further analyses. When a Si nanoparticle was covered by PANi, the color of the particle changed from light to dark brown (Fig. 1b). After the graphene and SP solution were mixed with assistance of the scCO_2_, the solution became a homogenous black (Fig. 1c). However, when ultrasonic treatment was applied instead of using the scCO_2_, a heterogeneous solution composed of brown and black particles was obtained, possibly representing the SP particles and graphene, respectively.

Many researchers have reported methods for the fabrication of Si/graphene composites, in which they prepared the composite using GO^5^,^15^,^19^,^20^,^21^. Because GO can be dispersed in water to form a stable suspension, the method using GO suspension is a convenient route to prepare homogeneous graphene-based composites without graphene agglomeration. Graphene nanosheets have a hydrophobic surface and show a tendency to stack and agglomerate in water or ethanol solvent. However, under a supercritical phase, the surface tension of the Si nanoparticles and graphene sheets is zero, enabling fabrication of a homogenous composite of hydrophilic and hydrophobic materials. When the CO_2_ was compressed, scCO_2_ easily diffuses into the layers of graphene sheets due to its low viscosity, high diffusivity, and small molecule size (Fig. 1d), generating repulsion in the free energy barrier that expands the gaps between the graphene sheets^22^. SP particles can be readily and rapidly intercalated between graphene sheets in scCO_2_. Then, CO_2_ expands and exits the reactor during the quick depressurization. SP particles can be deposited on graphene sheets instead of aggregating with each other or exiting with CO_2_ due to the π-π interactions between PANi and graphene sheets. When the depressurization is finished, graphene sheets decorated with SP particles are aggregated to form SPG composite in the ethanol solvent.

**Figure 2:**
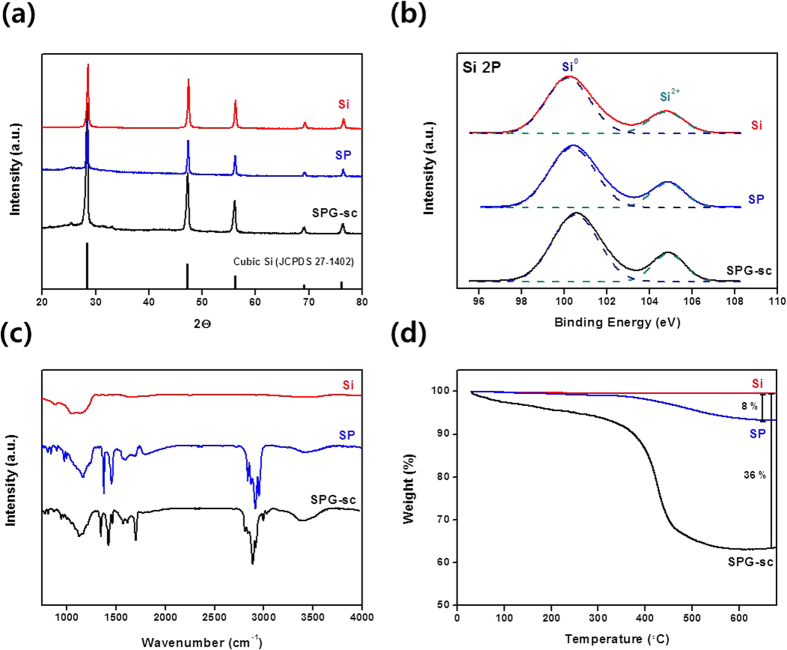
(**a**) XRD, (**b**) XPS of Si 2p orbitals, (**c**) FT-IR, and (**d**) TGA results of Si, SP, and SPG-sc.

The SPG-sc was characterized by X-ray diffraction (XRD), X-ray photoelectron spectra (XPS), Fourier-transform infrared spectroscopy (FT-IR), and thermo gravimetric analysis (TGA), as shown in Fig. 2. In the XRD spectrum of the bare Si, sharp peaks were observed at 28.4, 47.4, 56.2, 69.2, and 76.5 (Fig. 2a), which were attributed to crystalline-phase Si. These peaks were retained in SP and SPG-sc, indicating that the Si nanoparticles remained in the metallic state after the modification processes^30^,^31^. For an accurate analysis of Si, the X-ray photoelectron spectra in the Si 2p region were investigated (Fig. 2b). Two peaks centered at 100 and 105 eV were observed in the spectra of Si, SP, and SPG-sc, which are attributed to Si and SiO_2_, respectively^32^. Because a Si nanoparticle has a few nanometers of SiO_2_ layer on the surface (Figure S1), a peak from SiO_2_ was observed in the XPS spectrum. Note that the intensities of the Si and SiO_2_ peaks in the SPG-sc spectrum were the same as those in the bare Si spectrum, indicating that additional oxidation of the Si did not occur during the modification process.

The FT-IR spectrum of bare Si showed the characteristic peaks of partly oxidized Si, which were identified on the basis of the stretching vibration of the Si–O–Si bonds at 1,107 cm^−1^ (Fig. 2c). In the spectrum of SP, PANi can be identified by the C–H and C=C stretching vibrations of the benzoid and quinoid rings, which were seen at 2,989 cm^−1^ and 2,933 cm^−1^, respectively^33^,^34^. The amine groups of PANi were also detected from the N–H stretching at 3,303 cm^−1^ and the C–N stretching at 1,313 cm^−1^. These peaks were maintained in the SPG-sc spectrum, indicating that Si and PANi existed in the SPG-sc without any chemical deformation.

To measure the mass variation of the composite during the modification process, TGA was conducted in air within the temperature range of 30–700 °C (Fig. 2d). The PANi and graphene burned away in this temperature range, while the Si remained^35^. This implied that the weight loss of 8% in the SP spectrum was completely attributable to the PANi; thus, the contents of PANi and Si in the SP were calculated to be 8 and 92%, respectively. The SPG-sc spectrum showed a weight loss of 36%, which is attributed to the PANi and graphene. Considering the composition of SP, the contents of Si, PANi, and graphene in the SPG composite were calculated to be 64, 5.6, and 30.4%, respectively. Note that the content of graphene in SPG-sc was approximately 30%, which is identical to the graphene content before the process and indicates that the graphene was maintained during the process. Carbon dioxide has specific requirements to approach a supercritical phase (T_c_ = 31.1 °C and P_c_ = 73.8 bar); these conditions are much milder than those for other solvents such as water (T_c_ = 347 °C and P_c_ = 221 bar) or ethanol (T_c_ = 243 °C and P_c_ = 61 bar). Finally, it is possible to fabricate the composite without chemical or physical variation of the components.

**Figure 3:**
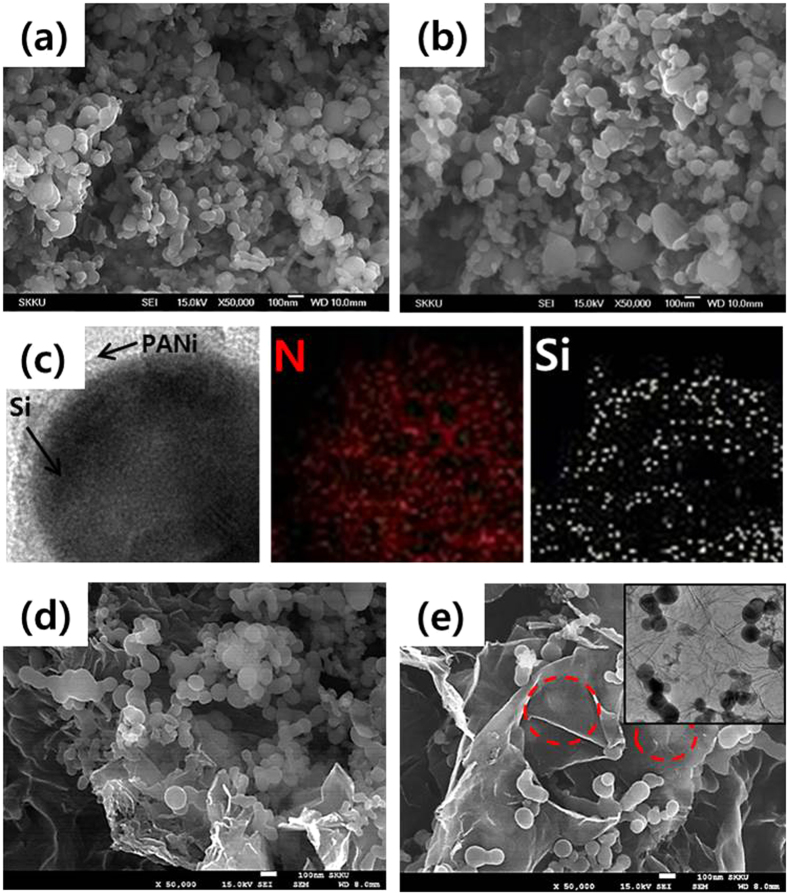
SEM images of (**a**) Si, (**b**) SP, (**d**) SPG-u, and (**e**) SPG-sc, and TEM images and the EDS maps of nitrogen and silicon. Inset of (**e**): TEM image of SPG-sc.

The morphology of the prepared samples was investigated by scanning electron microscopy (SEM). Si nanoparticles had spherical shapes with an average particle size of approximately 100 nm (Fig. 3a). After coating with PANi, SP particles maintained their original shape, but some of the particles were aggregated (Fig. 3b). Transmission electron microscopy (TEM) was conducted for accurate observation of SP. As shown in Fig. 3c, Si nanoparticles were homogeneously covered by a thin layer (~5 nm) of polyaniline. The morphologies of SPG were different depending on the fabrication method. SPG-u showed a cluster of nanoparticles and restacked graphene sheets (Fig. 3d), whereas SPG-sc showed graphene sheets with well-dispersed SP particles (Fig. 3e). The particles in SPG-sc were successfully deposited between graphene sheets and appeared as a bright sphere (red circle). In the SEM image under a low magnification, SPG-sc showed homogenous distribution of SP particles (Figure S2), and the TEM image showed that SP particles were deposited well on the graphene sheets even after ultrasonic energy was applied to disperse the particles in ethanol (inset of Fig. 3e). When bare Si nanoparticles (without PANi coating layer) were mixed with graphene nanosheets (Figure S3), particles were intercalated between the graphene sheets. However, most of the nanoparticles were aggregated, which might be due to the limited interaction between Si nanoparticles and graphene sheets.

**Figure 4:**
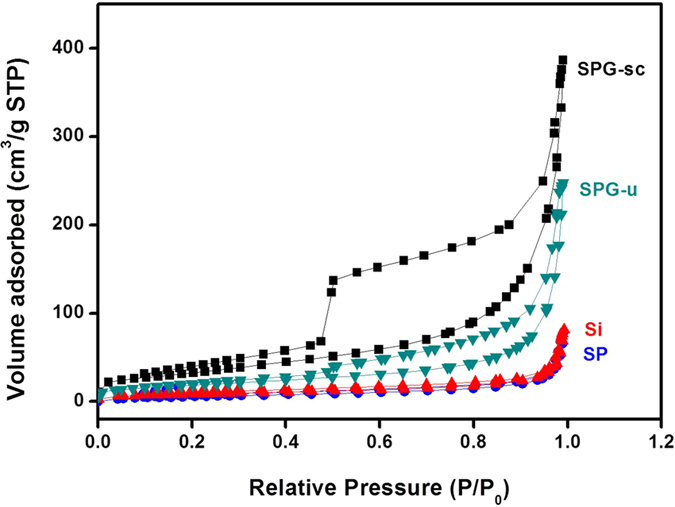
Nitrogen adsorption-desorption isotherms of the Si, SP, SPG-u, and SPG-sc electrodes.

To further investigate the morphology and its influence on physical properties, nitrogen adsorption-desorption analysis was conducted (Fig. 4). The nitrogen adsorption-desorption curves of the SPG composite exhibited a typical type IV isotherm with a hysteresis loop in the *P/P*_*0*_ range of 0.45–0.98, suggesting a porous structure of the composite. The BET specific surface area (S_E_) of the SPG composite was greater than 100 m^2^g^−1^, which might be due to the presence of graphene (S_E_ of graphene: 190 m^2^/g, Figure S4). The adsorption-desorption curves of the Si and SP showed negligible pores with a low S_E_ less than 25 m^2^/g. Despite having the same chemical composition of SPG, SPG-sc and SPG-u showed distinctly different physical properties. SPG-sc exhibited a surface area of 120 m^2^/g, which is much higher than the area of 65 m^2^/g for SPG-u. In SPG-sc, SP particles intercalated between graphene sheets could act as spacers, resulting in a higher surface area, whereas SP particles in SPG-u failed to intercalate. These results indicate that mixing under ethanol with the aid of scCO_2_ solvent is a rational approach for fabrication of well-dispersed SPG composites. Pore size distribution plots of the composites were obtained by the Barrett-Joyner-Halenda (BJH) method, showing that the pore volume of SPG-sc (0.635 cm^3^/g) was higher than that of SPG-u (0.278 cm^3^/g). Furthermore, plots of SPG composites showed wide distributions of pore size, from 2 nm to 30 nm (Figure S4). These results are quite different from those of the graphene composites in previous studies, but similar to that of bare graphene^19^,^36^. The graphene composites in previous studies tolerated heat treatment because of the necessity for reduction of graphene oxide or removal of organic components, which induced stacking of the graphene and accordingly removed the pores in the composite^37^. However, the SPGs did not undergo heat treatment and could therefore preserve pores with a wide range of diameters.

**Figure 5:**
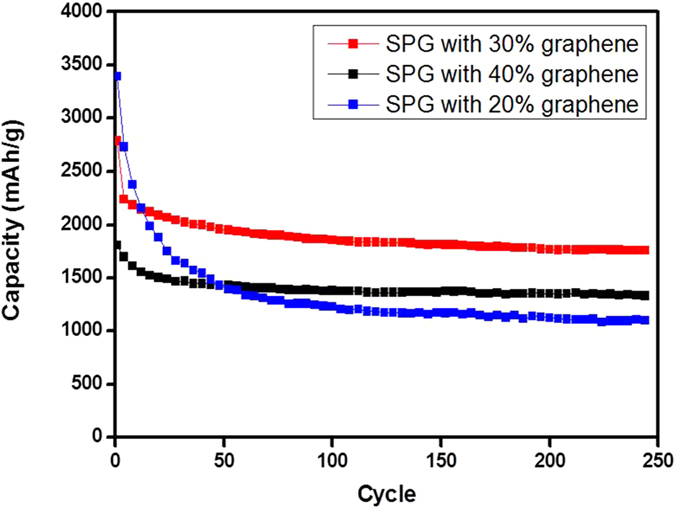
Specific capacity of the SPG-sc electrode according to the graphene content.

To characterize the electrochemical performance as anodes for lithium ion batteries, a galvanostatic charge-discharge test of the prepared electrodes was conducted at a current density of 400 mA/g in the voltage range of 0.01~3.0 V. As the content of graphene in the SPG-sc has a significant influence on the electrochemical properties, we prepared the SPG-sc samples with various graphene contents (Figure S5) and measured their specific capacity (Fig. 5). The SPG-sc electrode containing 20% graphene exhibited a high initial capacity of 2,714 mAh/g, but the capacity decayed rapidly with increasing cycles, reaching 1,099 mAh/g after 250 cycles. As the graphene content in the SPG-sc increased, the electrode exhibited enhanced cyclic retention with decreased initial capacity. Thus, the SPG-sc electrode with 40% graphene showed a capacity of only 1,331 mAh/g after 250 cycles due to its low initial capacity, even though the capacity only decreased slightly during cycling. The SPG-sc electrode with 30% graphene showed the highest capacity of 1,757 mAh/g after 250 cycles.

**Figure 6:**
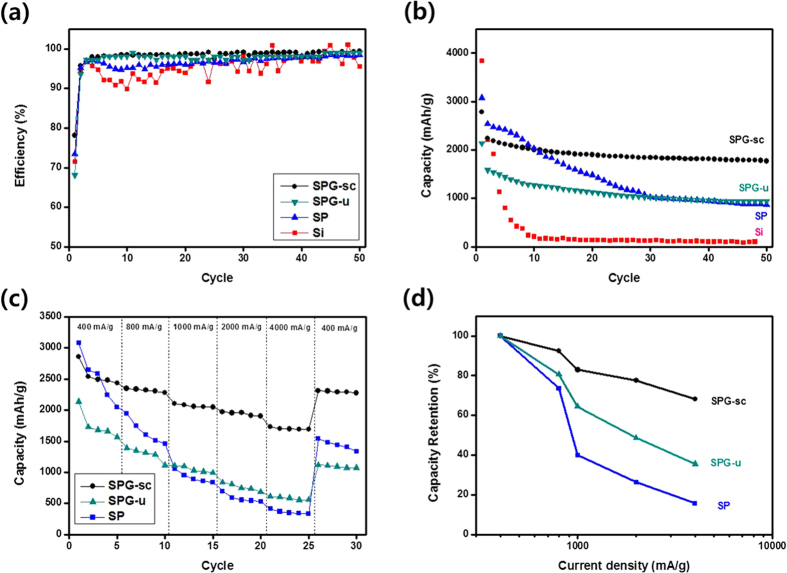
(**a**) Coulombic efficiency and (**b**) specific capacity of the Si, SP, SPG-u, and SPG-sc electrodes according to cycle number; (**c,d**) specific capacity of SPG-sc depending on applied current density.

To compare the cyclic performance of Si, SP, SPG-u, and SPG-sc electrodes, the coulombic efficiency and specific capacity were investigated according to cycle number (Fig. 6a,b). The values were calculated from the charge-discharge curves (Figure S6a). Charge-discharge curves of all samples exhibited voltage plateaus at 0.4 V for charging and 0.1 V for discharging, which are unique features of the alloying/dealloying reaction between crystalline Si and Li^38^. All samples showed a difference between charge and discharge capacity at the first cycle, associated with the formation of a solid electrolyte interface (SEI) layer with decomposition of the electrolyte^34^,^39^. The capacity difference decreased with increasing cycle number (Figure S6b), resulting in increasing coulombic efficiency. The coulombic efficiency values of the SPG electrodes rapidly increased from approximately 70% in the first cycle to 98% in the fourth cycle and remained above 99% during subsequent cycles (Fig. 6a). The efficiency values of Si and SP were lower than those of the SPG electrodes and showed unstable behavior even after 20 cycles. Si and SP electrodes might continuously undergo morphologic variation due to repeated volume expansion/contraction of Si particles during the cycles, inducing the additional formation of an SEI layer. At the first cycles, the Si electrode exhibited the highest specific capacity of 3,840 mAh/g compared with the specific capacity values of 3,077, 2,137, and 2,788 mAh/g for the SP, SPG-u, and SPG-sc electrodes, respectively (Fig. 6b). The SPG-sc electrode exhibited a similar capacity value to the SP electrode despite the lower content of Si, which reflects the enhanced electrical conductivity and ion accessibility owing to the presence of graphene sheets and enhanced surface area (Figure S7)^40^,^41^.

The specific capacity of the Si electrode decreased from 3,840 to 210 mAh/g after 10 cycles, and the specific capacity of the SP electrode decreased from 3,077 to 865 mAh/g after 50 cycles. In contrast, the specific capacity of the SPG-sc electrode reached 1,925 mAh/g after 50 cycles, demonstrating superior cycle performance. The specific capacity of the SPG-sc electrode remained almost constant after the 30th cycle, and the SPG-sc electrode exhibited the highest capacity of 1,757 mAh/g even after 250 cycles (Fig. 5), which is comparable to that reported for the Si/graphene-based composite (Table S1).

## Discussion

The dramatic capacity decay of the Si electrode is due to the destruction of the initial morphology during the cycles as a result of the volume change of Si (~300%), which causes Si nanoparticles to lose electrical contact from the electrode network^42^,^43^. In addition, morphologic variation of the Si electrode induces the additional formation of a SEI layer, which also increases the electrical resistance. In the case of the SPG-sc electrode, uniformly dispersed graphene sheets built an elastic framework that is favorable for electron transport. The elastic framework can tolerate the stress from the volume change of Si nanoparticles while maintaining its structure, which provides an efficient electrical path to the SP particles without the formation of an additional SEI layer (Figure S8). The porous structure of SPG-sc also contributed to preventing deformation of the framework structure because stresses are generated by the crushing of particles during volume expansion^44^. Pores in SPG provide space that might be filled by the expanded particles, preventing deformation of the structure. In fact, the thickness of the SPG-sc electrode increased from 23 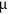
m to only 29 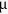
m after 50 cycles, whereas that of the Si electrode increased from 20 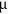
m to 51 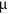
m. Expanded Si might fill the pores instead of pushing against each other; therefore, the thickness increase of the SPG-sc electrodes was much smaller than that of the Si electrode.

The PANi covering the Si particles also plays an important role in increasing the cycle stability. The Si/graphene composite prepared using scCO_2_ showed a capacity of 1531 mAh/g after 50 cycles (Figure S9), which is a slightly lower value than that of SPG-sc. SP particles and graphene could participate in π-π interactions, which induce the well distributed SP particles on the graphene sheets and provide a stable electrical path between the SP particles and the graphene framework^45^. Moreover, elastic PANi releases the stress formed during volume expansion of the Si^29^,^30^. Thus, PANi plays an important role in creating and retaining high electronic conductivity without escape from the Si during charge or discharge, which was confirmed by comparing EDS results of the electrode before and after cycling (Figure S8). When we conducted cycle tests with SPG-sc after thermal treatment, the electrode showed a deteriorated cycle performance and morphology of cracked film (Figure S10). During the thermal treatment, stacking of graphene and pyrolysis of PANi might occur, resulting in a decrease in porosity of the SPG and elasticity of the PANi shell. This result confirms the advantage of the absence of thermal treatment and the importance of the porous structure and elasticity of a PANi shell.

As rate capability is an important parameter in evaluating lithium battery electrodes, we investigated those of the SP and the SPG composites (Fig. 6c,d). The rate capability of the SPG-sc electrode outperformed that of the SP and SPG-u electrodes. When 4,000 mA/g was applied to the SPG-sc, it still delivered a capacity above 1,690 mAh/g, and recovery of the capacity was confirmed when the current returned to 400 mA/g. Also, the SPG-sc showed stable cyclic behavior when a current density of 4000 mA/g was applied (Figure S11). The rate capability of SPG-sc was comparable to that of SP and to that of the Si-based materials described previously (Table S1). Recently, Nam *et al*. reported that a porous graphene composite exhibited a higher rate performance than bare graphene composite, in which the porous structure could provide efficient paths for lithium ions^45^. Finally, the SPG-sc electrode exhibited enhanced rate capability and cyclic stability thanks to the porous structure.

In summary, homogenous Si/graphene composite was synthesized with the assistance of a scCO_2_ fluid following chemical polymerization of polyaniline. The composite was prepared under mild temperatures in a short time due to the use of scCO_2_ fluid. The Si particles were homogenously deposited between graphene sheets, and the components in the composite, including Si and polyaniline, maintained their properties. The composite showed a large surface area of 120 m^2^/g and high electrical conductivity. The composite exhibited high cycle performance (1,789 mAh/g after 250 cycles) and rate capability (1,690 mAh/g at a current density of 4,000 mA/g), which is attributed to the buffering of the elastic graphene framework and polyaniline shell. In addition, the porous structure provides additional space that is filled by expanded particles and efficient paths for the lithium ions, which are also critical factors for the improved cycle and rate performance.

## Methods

### Materials

Si nanoparticles (average particle size of ~100 nm) and poly(acrylic acid) (PAA; Mw, 250,000, 35 wt % in H_2_O) were obtained from Alfa Aesar. Aniline, ammonium persulfate (APS; (NH_4_) _2_S_2_O_8_), and hydric acid (HCl; 35%) were purchased from Aldrich. Graphene nanosheets (model: xGnP) of sub-micrometer to 100 lm in width and 1 nm to 15 nm in thickness were obtained from XG Sciences, Inc., Michigan, USA.

### Preparation of SP and SPGs

SP particles were synthesized by a chemical polymerization method. Briefly, 0.1 g of Si nanoparticles was dispersed in 100 mL of ethanol in an ultrasonic bath for 30 min, and 0.1 mL of aniline was added to the solution. After ultrasonication for 30 min to fully disperse the aniline monomer in ethanol, 4.0 mL of HCl aqueous solution containing 0.6 g of APS was added to the above ethanol solution. The mixture was continuously stirred for another 6 h, forming the PANI shell. The product was washed several times with ethanol and dried at 60 °C for 12 h.

To obtain SPG-sc, 0.7 g of SP particles and 0.3 g of graphene nanosheets were dispersed in 20 mL of ethanol using an ultrasonic bath for 30 min. The dispersion was loaded into a high-pressure 200 mL vessel. After the autoclave was sealed, the vessel was charged with CO_2_ to 14 MPa at 60 °C and maintained for 30 min with stirring, after which the vessel was decompressed by the emission of CO_2_. The solution was dried at 80 °C to obtain a powder. To obtain SPG-u, the dispersion was mixed using a tip (bar type) sonication instrument at 750 W for 1 h instead of using CO_2_. Also, SPG composite was also prepared by mechanical stirring, in which SP particles were not well dispersed (Figure S12); thus, further analyses were not conducted.

### Characterization

The morphology of the products was observed using scanning electron microscopy (SEM; JSM7600, JEOL) and transmission electron microscopy (HR-TEM; JEM ARM 200F). The crystal structure and chemical composition were characterized using X-ray diffractometry (XRD, D8 Advance) and X-ray photoelectron spectroscopy (XPS; ESCA2000, VG Microtech), respectively. Functional groups on the surface of the samples were characterized using a Fourier-transform infrared spectrometer (FT-IR; Bruker tensor 27), and the Brunauer−Emmett−Teller surface area was measured using a Belsorp-mini II apparatus (BEL Inc., Japan).

### Measurement of Electrochemical Properties

Electrochemical properties of the prepared samples were measured using a 2032 coin-type cell. The active material (70 wt %), PAA as a binder (15 wt %), and carbon black as a conductor (15 wt %) were carefully mixed in a water/ethanol solvent. The slurry was then cast on Cu foil and dried at 80 °C under a vacuum for 12 h to remove the solvent. The electrode film was punched into 15-mm-diameter discs (area 1.77 cm^2^) and weighed. The cell was fabricated in a glove box filled with high-purity argon gas using 1 M LiPF_6_ dissolved in ethylene carbonate/dimethyl carbonate/ethylmethyl carbonate as the electrolyte (volume ratio of EC/DMC/EMC = 1:1:1). The charge and discharge behaviors were monitored using a battery test system (WonATech Corp., Korea) with a voltage range of 0.005–3.0 V (vs. Li/Li+) at room temperature. Electrical impedance spectroscopy (EIS) measurements were conducted using a potentiostat (VSP, Princeton Applied Research, USA), applying a 10 mV amplitude sine wave in the frequency range of 0.1 Hz to 100 kHz.

## Additional Information

**How to cite this article**: Lee, S. H. *et al*. Supercritical Carbon Dioxide Assisted Process for Well-Dispersed Silicon/Graphene Composite as a Li ion Battery Anode. *Sci. Rep.* **6**, 32011; doi: 10.1038/srep32011 (2016).

## Supplementary Material

Supplementary Information
