# Supplementary material for: Supercritical Carbon Dioxide-Assisted Process for Well-Dispersed Silicon/Graphene Composite as a Li ion Battery Anode
Source: Sci Rep. 2016 Aug 18;6:32011. doi: 10.1038/srep32011 (PMC4989224; doi:10.1038/srep32011)
Supplement: Supplementary Information [file srep32011-s1.pdf]

Supporting Information

Supercritical Carbon Dioxide-Assisted Process for Well-Dispersed Silicon/Graphene Composite as a Li ion Battery Anode

Sang Ha Lee[1], Sengyoen Park[1], Min Kim[1], Dohyeon Yoon[2], Chalathorn Chanthad[1],

Misuk Cho[1], Jaehoon Kim[2], Jong Hyeok Park[3], Youngkwan Lee[1*]

[1]School of Chemical Engineering

Sungkyunkwan University, 440-746 Suwon, Korea

[1]School of Mechanical Engineering

Sungkyunkwan University, 440-746 Suwon, Korea

[2]Department of Chemical and Biomolecular Engineering

Yonsei University, 440-746 Suwon, Korea

*Correspondence: yklee@skku.edu

Tel.: +82-31-290-7259

Fax: +82-31-299-4711

Figures (supporting information)

Figure S1. TEM image of (a) bare Si, (b) SP, and (c) SP after $scCO_2$ treatment.

Figure S2. SEM images under a low magnification, and their mapping results: (a) SPG-sc and (b) SPG-u.

Figure S3. SEM image of Si/graphene composite prepared using $scCO_2$.

Figure S4. (a) Nitrogen adsorption-desorption isotherms, and (b) pore distribution plot of graphene and SPG composites.

Figure S5. SEM image of SPG-sc with (a) 20%, (b) 30%, and (c) 40% graphene contents.

Figure S6. Charge-discharge curves (a) of the Si, SP, SPG-u, and SPG-sc electrodes at first cycle and (b) of the SPG-sc electrode according to cycle number.

Figure S7. EIS spectra of the Si, SP, SPG-u, and SPG-sc electrodes after the first cycle.

Figure S8. (a) EIS spectra and (b) SEM images with EDS results of the SPG-sc electrode after 1 and 100 cycles.

Figure S9. Specific capacities of SPG and the Si/graphene electrode according to cycle number (both composites were prepared using $scCO_2$ fluid).

Figure S10. (a) Specific capacities of the electrode consisting of SPG-sc with/without thermal treatment according to cycle number, and (b) SEM image of the SPG-sc electrode with thermal treatment after cycling.

Figure S11. Specific capacities of SPG-sc at a current density of 4000 mA/g. (First three cycles of the test were conducted at a current density of 400 mA/g)

Figure S12. (a) SEM image and (b) nitrogen adsorption-desorption isotherm of SPG composite prepared with stirring.

Table S1. Comparison of the performance of Si/graphene-based electrodes.

Figures (supporting information)

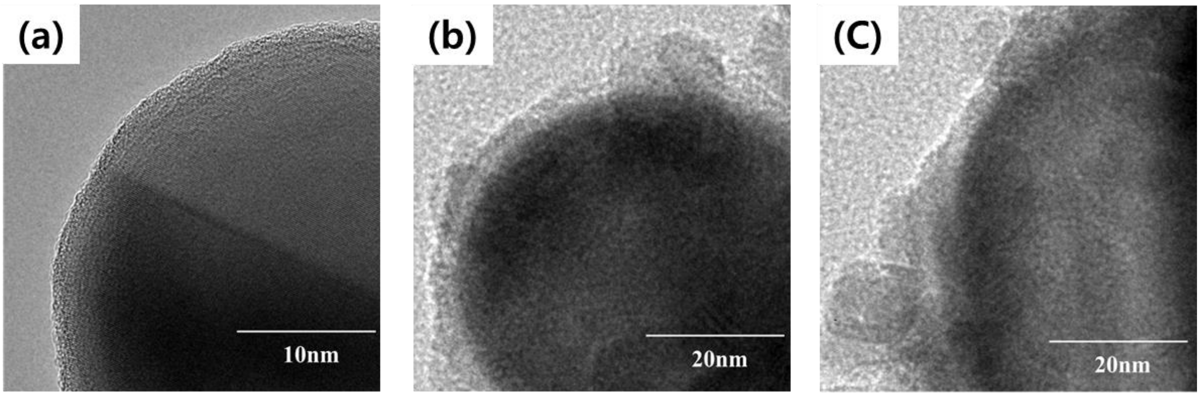

Figure S1. TEM image of (a) bare Si, (b) SP, and (c) SP after $scCO_2$ treatment.

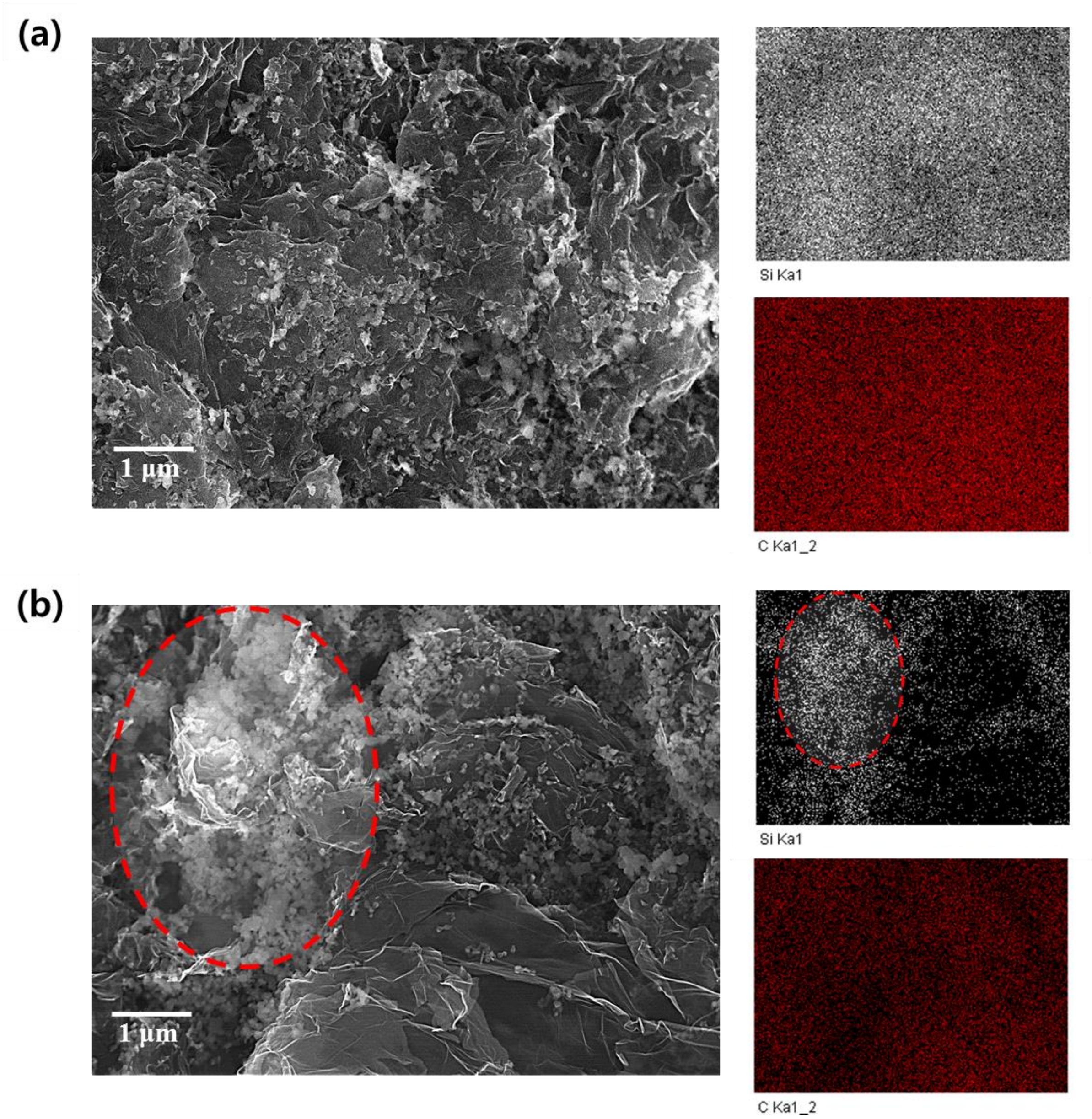

Figure S2. SEM images under a low magnification, and their mapping results: (a) SPG-sc and (b) SPG-u.

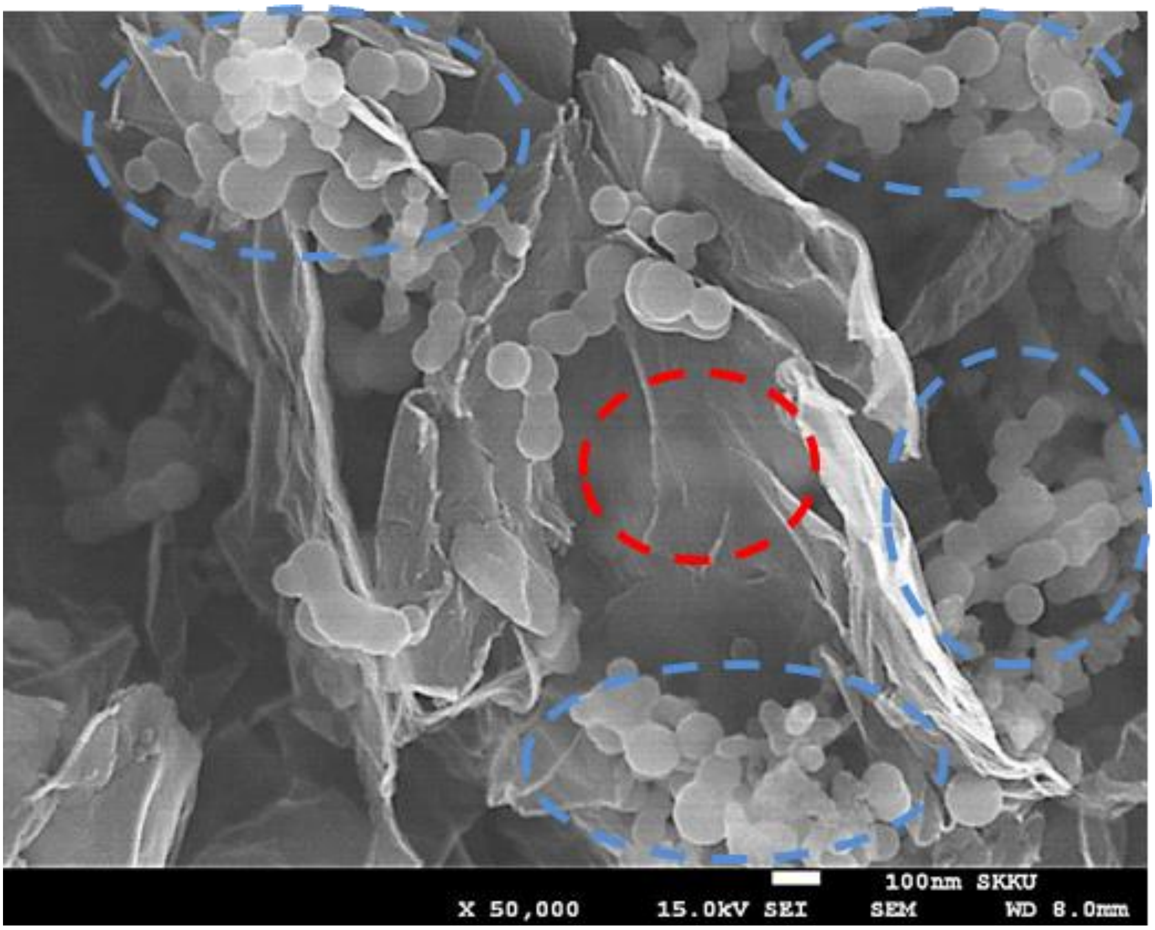

Figure S3. SEM image of Si/graphene composite prepared using $scCO_2$.

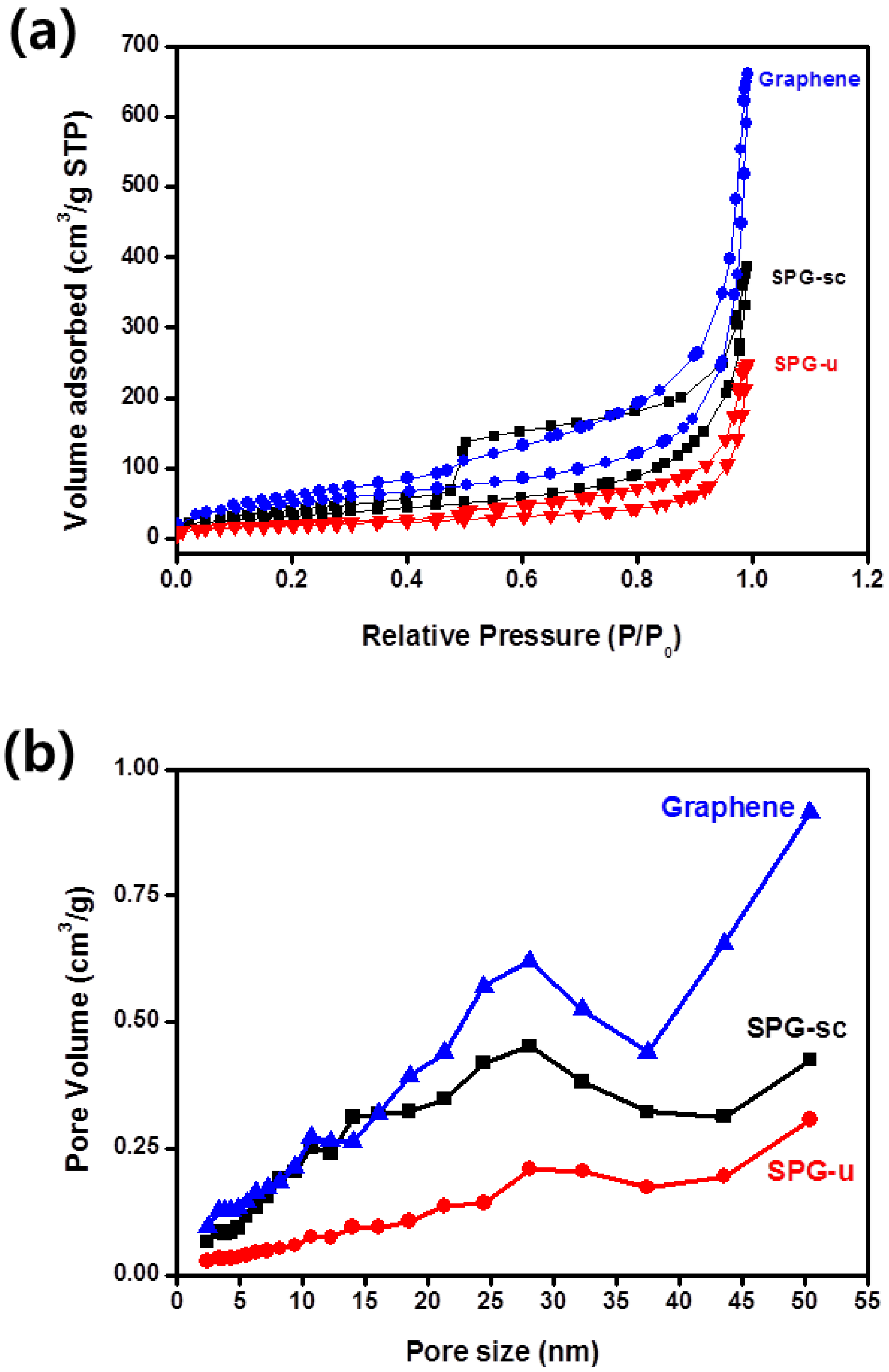

Figure S4. (a) Nitrogen adsorption-desorption isotherms, and (b) pore distribution plot of graphene and SPG composites.

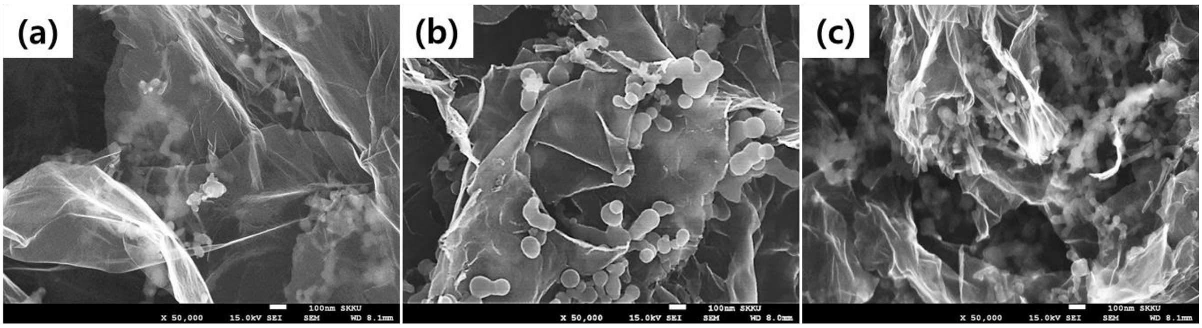

Figure S5. SEM image of SPG-sc with (a) 20%, (b) 30%, and (c) 40% graphene contents.

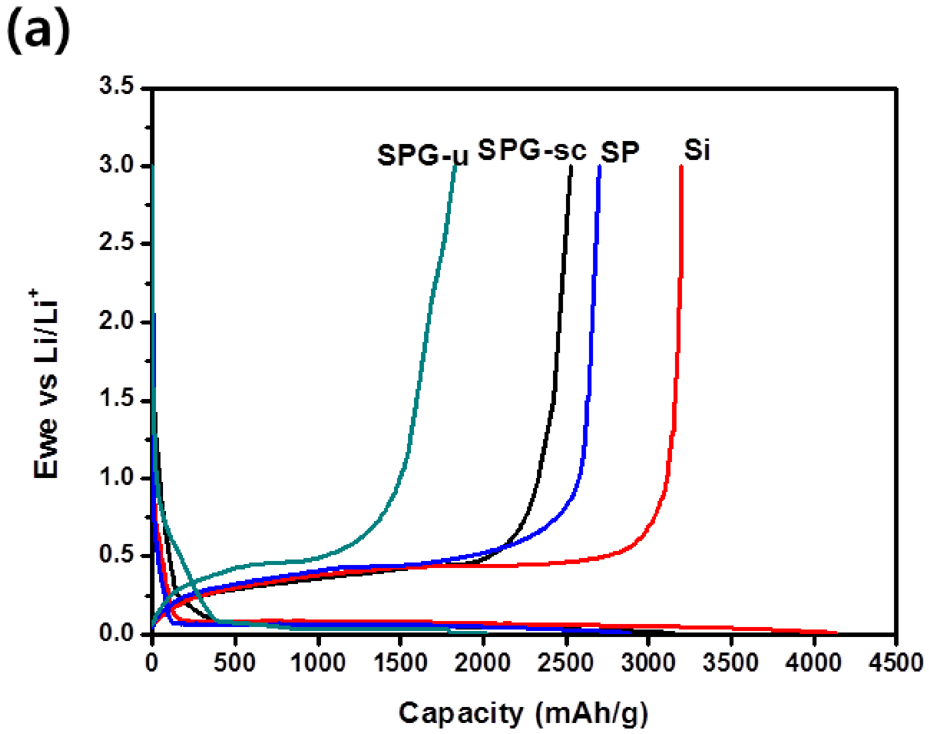

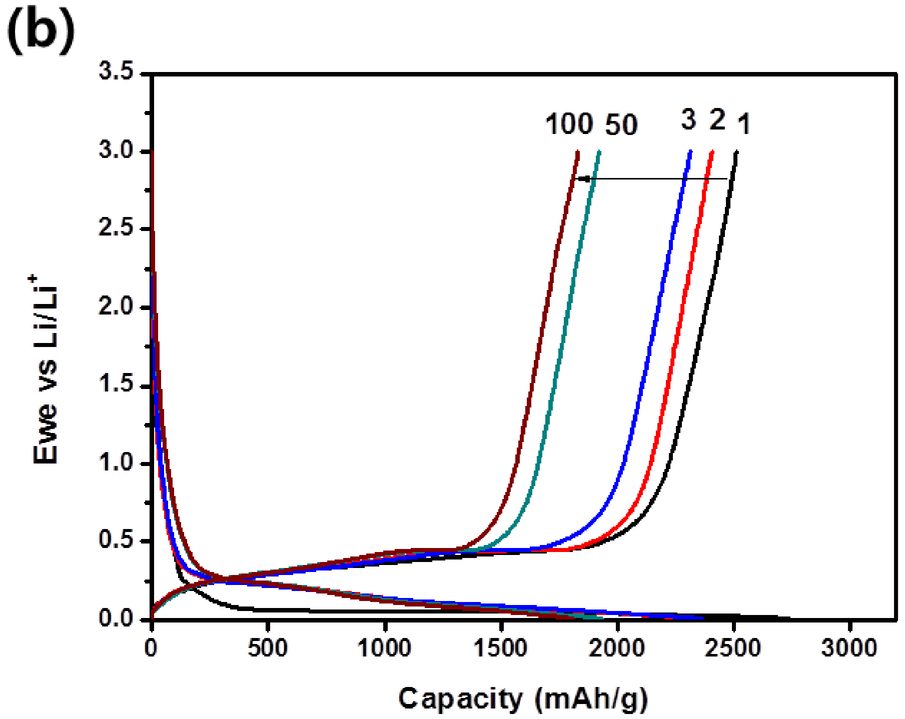

Figure S6. Charge-discharge curves (a) of the Si, SP, SPG-u, and SPG-sc electrodes at first cycle and (b) of the SPG-sc electrode according to cycle number.

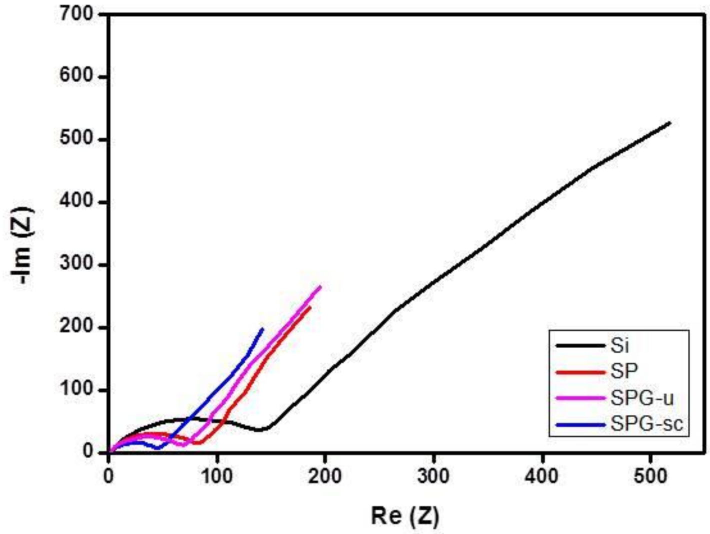

Figure S7. EIS spectra of the Si, SP, SPG-u, and SPG-sc electrodes after the first cycle.

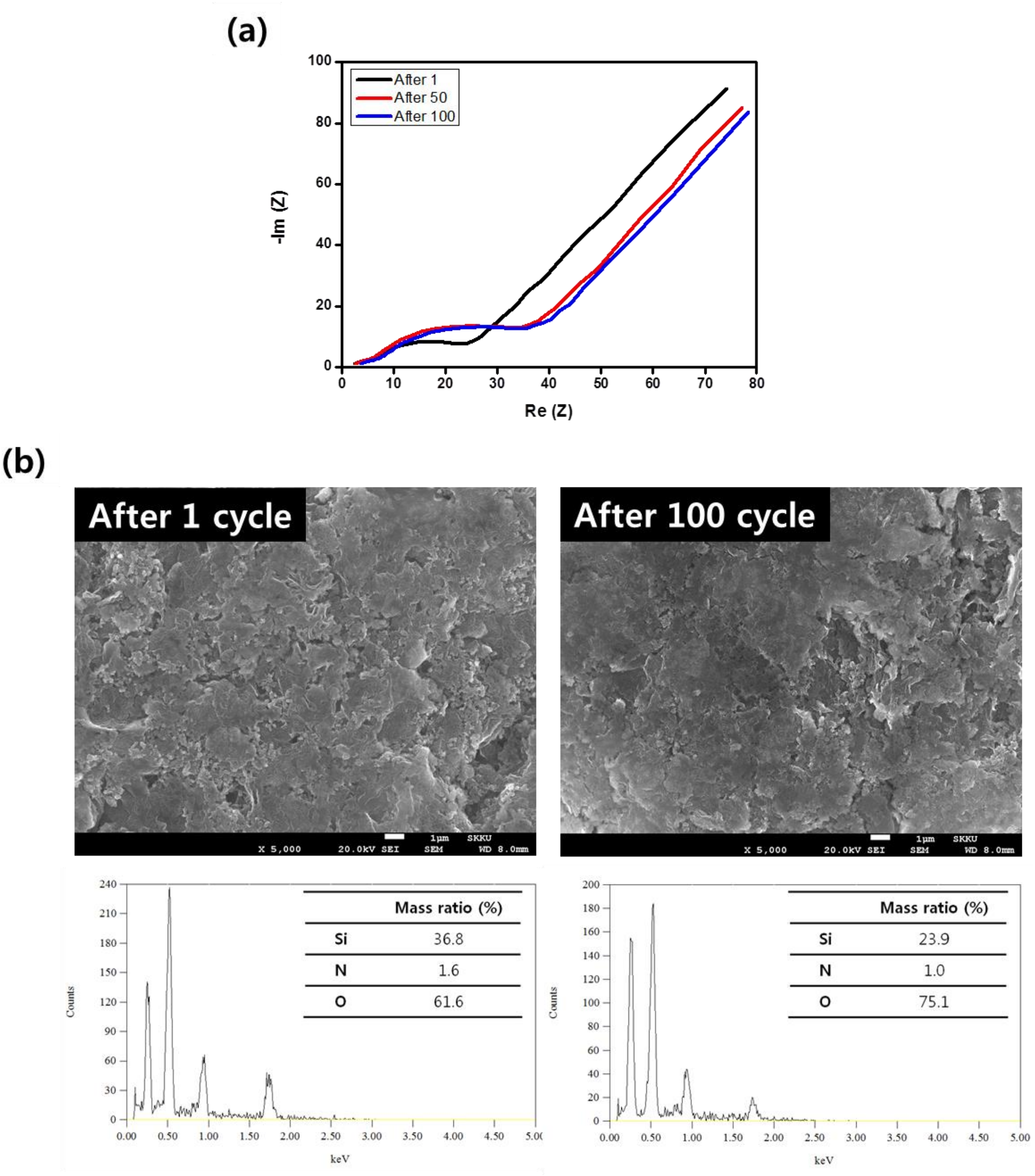

Figure S8. (a) EIS spectra and (b) SEM images with EDS results of the SPG-sc electrode after 1 and 100 cycles.

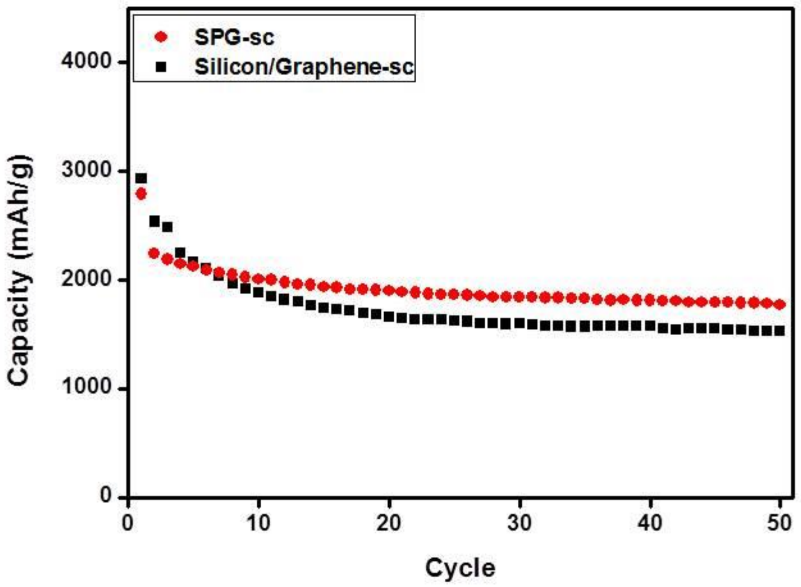

Figure S9. Specific capacities of SPG and the Si/graphene electrode according to cycle number (both composites were prepared using scCO_2 fluid).

(a)

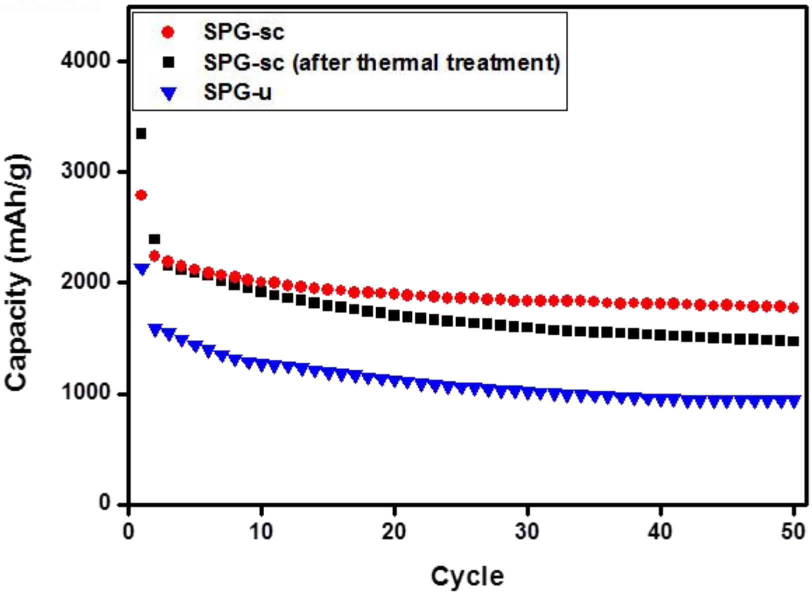

(b)

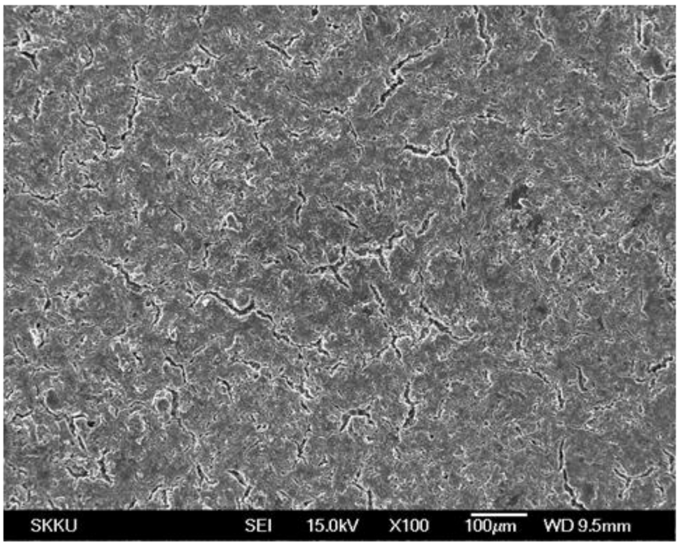

Figure S10. (a) Specific capacities of the electrode consisting of SPG-sc with/without thermal treatment according to cycle number, and (b) SEM image of the SPG-sc electrode with thermal treatment after cycling.

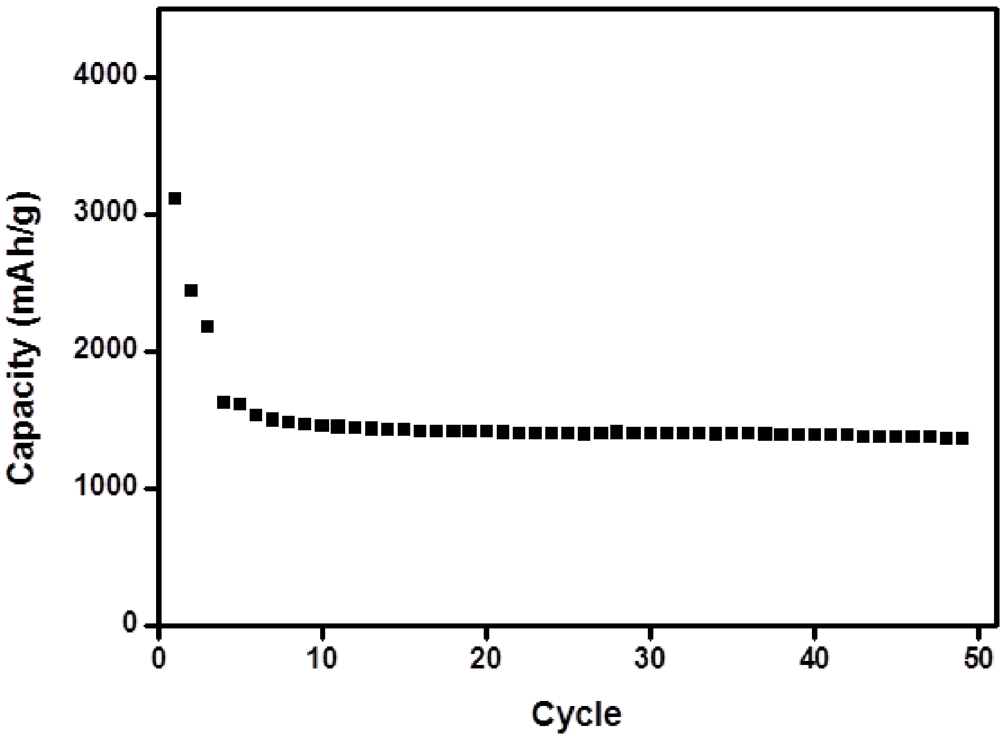

Figure S11. Specific capacities of SPG-sc at a current density of 4000 mA/g. (First three cycles of the test were conducted at a current density of 400 mA/g)

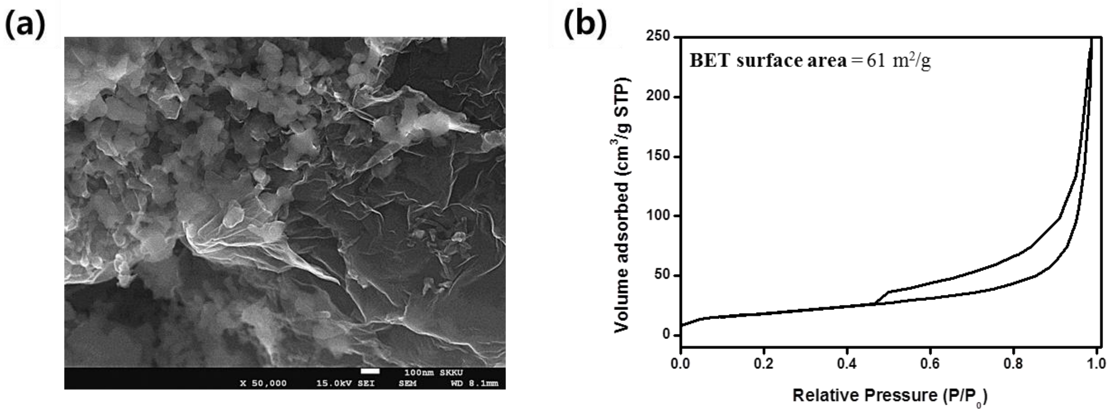

Figure S12. (a) SEM image and (b) nitrogen adsorption-desorption isotherm of SPG composite prepared with stirring.

Table S1. Comparison of the performance of Si/graphene-based electrodes.

Reference	Materials	Specific capacity mAh/g (cycle number, current density)	Specific capacity at high rate mAh/g (current density)
**1**	Si/rGO	1205 (150, 100mA/g)	990 (1600mA/g)
**2**	Si-carbon NW /rGO	1280 (100, 2100mA/g)	1600 (2100mA/g)
**3**	Si/carbon/rGO	579.6 (100, 500mA/g)	-
**4**	Si/Graphene	1071 (200, 0.4mA/cm^2)	534 (2mA/cm^2)
**5**	Si/rGO	1434 (100, 500mA/g)	1220 (5000mA/g)
**6**	Si/silver/Graphene	975 (100, 0.2C)	≒800 (1C)
**7**	Si/Carbon/rGO	650 (200, 1000mA/g)	715 (1500mA/g)
**8**	Si/Ti_2O_3/rGO	985 (100, 100mA/g)	≒1100 (10000mA/g)
**9**	Si-pyrolyzed PANi/rGO	900 (300, 2000mA/g)	1184 (2000mA/g)
**10**	Porous Si/rGO	1942 (100, 100mA/g)	1521 (4000 mA/g)
**11**	Si/rGO (binder-free)	1500 (1000, 100mA/g)	1552 (1C)
**12**	Si/graphene (binder-free)	1353.8 (150, 50mA/g)	412 (8000mA/g)
**This work**	**Si/PANi/Graphene**	**1784 (250, 400mA/g)**	**1690 (4000 mA/g)**

References

1 Zhou, X., Yin, Y.-X., Wan, L.-J. & Guo, Y.-G. Self-Assembled Nanocomposite of Silicon Nanoparticles Encapsulated in Graphene through Electrostatic Attraction for Lithium-Ion Batteries. *Advanced Energy Materials* **2**, 1086-1090, doi:10.1002/aenm.201200158 (2012).

2 Wang, B. *et al.* Adaptable silicon–carbon nanocables sandwiched between reduced graphene oxide sheets as lithium ion battery anodes. *ACS nano* **7**, 1437-1445 (2013).

3 Chae, C., Noh, H.-J., Lee, J. K., Scrosati, B. & Sun, Y.-K. A High-Energy Li-Ion Battery Using a Silicon-Based Anode and a Nano-Structured Layered Composite Cathode. *Advanced Functional Materials* **24**, 3036-3042, doi:10.1002/adfm.201303766 (2014).

4 Hu, R., Sun, W., Chen, Y., Zeng, M. & Zhu, M. Silicon/graphene based nanocomposite anode: large-scale production and stable high capacity for lithium ion batteries. *Journal of Materials Chemistry A* **2**, 9118, doi:10.1039/c4ta01013b (2014).

5 Liu, X. *et al.* Understanding the Effect of Different Polymeric Surfactants on Enhancing the Silicon/Reduced Graphene Oxide Anode Performance. *The Journal of Physical Chemistry C* **119**, 5848-5854, doi:10.1021/jp512152f (2015).

6 Gu, M. *et al.* Double locked silver-coated silicon nanoparticle/graphene core/shell fiber for high-performance lithium-ion battery anodes. *Journal of Power Sources* **300**, 351-357, doi:10.1016/j.jpowsour.2015.09.083 (2015).

7 Zhang, F. *et al.* Pyrolytic carbon-coated Si nanoparticles on elastic graphene framework as anode materials for high-performance lithium-ion batteries. *Carbon* **82**, 161-167, doi:10.1016/j.carbon.2014.10.046 (2015).

8 Park, A. R. *et al.* Si/Ti2O3/Reduced Graphene Oxide Nanocomposite Anodes for Lithium-Ion Batteries with Highly Enhanced Cyclic Stability. *ACS Appl Mater Interfaces* **7**, 18483-18490, doi:10.1021/acsami.5b04652 (2015).

9 Li, Z. F. *et al.* Novel pyrolyzed polyaniline-grafted silicon nanoparticles encapsulated in graphene sheets as Li-ion battery anodes. *ACS Appl Mater Interfaces* **6**, 5996-6002, doi:10.1021/am501239r (2014).

10 Feng, J. *et al.* Chemical dealloying synthesis of porous silicon anchored by in situ generated graphene sheets as anode material for lithium-ion batteries. *Journal of Power Sources* **287**, 177-183, doi:10.1016/j.jpowsour.2015.04.051 (2015).

11 Chang, J. *et al.* Multilayered Si nanoparticle/reduced graphene oxide hybrid as a high-performance lithium-ion battery anode. *Adv Mater* **26**, 758-764, doi:10.1002/adma.201302757 (2014).

12 Li, N., Jin, S., Liao, Q., Cui, H. & Wang, C. X. Encapsulated within graphene shell silicon nanoparticles anchored on vertically aligned graphene trees as lithium ion battery anodes. *Nano Energy* **5**, 105-115, doi:10.1016/j.nanoen.2014.02.011 (2014).
